# Engineering Organ Conformality for Precise Intracellular Delivery

**DOI:** 10.34133/research.1284

**Published:** 2026-05-07

**Authors:** Yanling Hu, Dongliang Yang, Zhen Yang

**Affiliations:** ^1^ College of Life and Health, Nanjing Polytechnic Institute, Nanjing, China.; ^2^ Key Laboratory of Flexible Electronics (KLOFE) and Institute of Advanced Materials (IAM), School of Physical and Mathematical Sciences, Nanjing Tech University (NanjingTech), Nanjing, China.; ^3^ Strait Institute of Flexible Electronics (SIFE, Future Technologies), Fujian Key Laboratory of Flexible Electronics, Fujian Normal University and Strait Laboratory of Flexible Electronics (SLoFE), Fuzhou 350117, China.

## Abstract

Bioelectronic patches have emerged as promising tools for localized and precise intracellular delivery. However, their application is limited by poor conformability and suboptimal delivery efficiency on anatomically complex organs. A recent study published in *Cell* introduced POCKET, a kirigami-based electrotransfection bioelectronic patch for conformable intracellular delivery on complex organ surfaces. This study establishes a universal conformality framework for maximizing organ coverage. POCKET connects nanopores to target cells, enhancing electroporation and transmembrane transport for efficient drug delivery to organs. By integrating mechanical conformality with interfacial bioelectronics, this platform enhances spatial precision and therapeutic efficacy in complex organs, promising advances in gene therapy, organ protection, and regenerative medicine.

Efficient intracellular delivery in vivo remains a major challenge in precision medicine. Although diverse approaches have been developed, their performance often sharply declines when delivery occurs locally on intact organs with curved, irregular, and dynamically moving surfaces [1–4]. In these settings, the main limitation is poor tissue–device contact, which causes uneven electric field distribution, lower delivery efficiency, and limited spatial control [5,6]. Traditional delivery platforms, including ultrasound-, optical-, and electric-mediated systems, are often hindered by off-target effects, interface mismatch, and device size, compromising the precise treatment of complex organs [7–9]. Even flexible biopatches improve tissue adaptability but fail to achieve uniform, extensive contact on complex organ surfaces [10–13].

Recently, Chang et al. [14] identified organ conformality as a key factor for intracellular delivery, shifting focus from patch flexibility to quantitative interface engineering [14]. By integrating kirigami technique and conformality theory [15,16], the wireless, implantable POCKET patch adapts to different organ shapes while ensuring full surface coverage. Under interfacial adhesion constraints, POCKET achieves wrinkle- and delamination-free full conformality on complex curved surfaces from cellular to organ scales. In the conformal state, nanopores in the POCKET align with cells, ensuring uniform curved-surface electric distribution to create a stable and large-area electroporation interface for drug delivery. For example, on a porcine ovary, optimized geometric parameters allowed the kirigami patch to achieve 97% wrinkle-free conformal coverage, compared to 40% for a traditional planar patch. Moreover, with 49% fracture strain and 0.9-MPa modulus, POCKET seamlessly integrates with dynamic organ surfaces. In general, POCKET’s normal-operation deformation (6% to 12%) remains far below the failure threshold, ensuring a stable functional interface. Thus, POCKET’s kirigami design shifts from passive to active adaptation, thereby enabling precise delivery on complex and dynamic biological surfaces.

The structural design of POCKET is matched by its engineered functional interface. Unlike flat, mesh, or hydrogel patches, POCKET combines customizable conformability with electroporation, offering superior scalability. On complex curved surfaces, flat and hydrogel patches wrinkle and deliver unevenly, while stretchable mesh patches lose functional continuity and tissue contact. POCKET with electrotransfection function features a 4-layer functional architecture comprising a polydimethylsiloxane (PDMS) film, a silver nanowire (AgNW) electrode, a drug-loaded hydrogel layer, and a nanopore polyethylene terephthalate (PET) film containing a wireless powering module (Fig. 1A). The high-resistance nanopores concentrate the electric field locally at the cell membrane, generating a transmembrane potential that enables wireless-driven reversible electroporation (Fig. 1B) [17]. Electroporation enables POCKET to deliver therapeutics directly to the cytosol, bypassing endocytosis to boost efficiency [17]. The results indicated that POCKET achieved ~80% transfection efficiency at 20 V and a 10-fold enhancement in drug delivery speed compared to systems without nanopores [14]. These findings reveal a key principle for future electronic patch: Interface architecture can be as critical as electrical input itself.

**Fig. 1.**
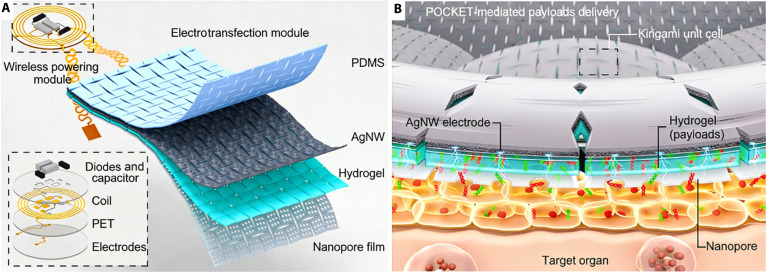
(A) Anatomical and (B) drug delivery mechanism diagram of POCKET. The nanopore–cell interface enhances the local electric field and accelerates electroporation transport for intracellular delivery. Reproduced from [14] with permission from Elsevier Ltd., copyright 2026.

The biological applications highlight the platform’s significance. In preclinical ovarian gene therapy, the POCKET system targeted delivery of breast cancer gene 1 (BRCA1) plasmid to ovarian surface epithelial cells. These transfected cells spread the therapeutic effect throughout the organ by secreting extracellular vesicles carrying plasmid-derived mRNA. In a BRCA1-deficient mouse model, this treatment markedly reduced DNA damage and restored ovarian function and fertility to wild-type levels. Compared with conventional approaches such as lipid nanoparticles injection, the POCKET system achieved a 3.4- to 7.4-fold higher transfection efficiency [14]. During treatment, ovarian cancer incidence fell from 100% to 0, showing the system’s preventive potential. This achievement avoids exogenous DNA integration in germ cells, eliminating gene therapy’s reproductive risk and providing a safe and translatable strategy for gene therapy to reproductive organs. In renal ischemia–reperfusion injury, POCKET enabled localized, sustained dexamethasone delivery to the kidney surface, expanding the platform’s therapeutic scope beyond gene transfer. Compared with oral administration, this approach enhanced renal drug exposure by 2.4-fold and reduced systemic exposure by 99.5% [14], a redistribution that benefits therapies limited by off-target toxicity. By concentrating therapeutic effect at the injury site, the platform reduced renal tubular damage and fibrosis while avoiding adverse effects of systemic dexamethasone exposure, including osteoporosis and infection risk. Thus, POCKET functions as both a bioelectronic gene delivery system and a versatile, safe platform for organ-restricted pharmacotherapy.

Despite progress with the POCKET platform, several challenges remain for the translation of organ-conformal bioelectronic delivery. For large human organs, the multielectrode configuration requires systematic optimization—e.g., modular electrode management ensures uniform field distribution for controllable local or whole-organ drug delivery [18]. To avoid secondary removal surgery and tissue damage, a biodegradable POCKET is crucial, e.g., using materials such as poly(lactide-*co*-glycolide), polycaprolactone, or gelatin hydrogel. Meanwhile, its mechanical properties should match those of the contacting tissue, and its degradation rate must align with the treatment duration [19]. In addition, the therapeutics loading, release, and electrophysiological mechanisms require investigation [20]. Manufacturing repeatability, equipment standardization, and clinical workflow integration are also crucial for future translation. These challenges do not diminish the theoretical value but highlight next steps for advancing organ-conformal bioelectronic therapies.

This research presents a flexible patch, identifies, and solves core bottlenecks in local intracellular delivery. Using kirigami conformality and nanopore electrodelivery, POCKET efficiently and tightly adheres to organs to improve drug delivery to cells. The future progress in bioelectronic medicine may rely less on soft devices and more on anatomical design, controllable activation, and targeted delivery. Further scaling up POCKET requires application-specific wireless power and electrode management. Meanwhile, integration of biosensors will enable visualized, smart, real-time, on-demand treatment for chronic diseases (e.g., diabetes, inflammation, and hypertension). With these challenges resolved, POCKET will provide a powerful delivery platform for precision intervention in complex organs.
